# Effect of four-week cannabidiol treatment on cognitive function: secondary outcomes from a randomised clinical trial for the treatment of cannabis use disorder

**DOI:** 10.1007/s00213-022-06303-5

**Published:** 2023-01-04

**Authors:** Rachel Lees, Lindsey A. Hines, Chandni Hindocha, Gianluca Baio, Natacha D. C. Shaban, George Stothart, Ali Mofeez, Celia J. A. Morgan, H. Valerie Curran, Tom P. Freeman

**Affiliations:** 1 Addiction and Mental Health Group, Department of Psychology, University of Bath, Bath, UK; 2 Department of Psychology, University of Bath, Bath, UK; 3 Population Health Science, Bristol Medical School, University of Bristol, Bristol, UK; 4 Clinical Psychopharmacology Unit, UCL, London, UK; 5 Department of Statistical Science, UCL, London, UK; 6 University of Oxford, Oxford, UK; 7 Pain Management Centre, National Hospital for Neurology and Neurosurgery, UCLH, London, UK; 8 Psychopharmacology and Addiction Research Centre, University of Exeter, Exeter, UK

**Keywords:** Cannabidiol, Cannabis use disorder, Clinical trial, Cognition, Verbal learning and memory

## Abstract

**Rationale:**

Chronic cannabis use is associated with impaired cognitive function. Evidence indicates cannabidiol (CBD) might be beneficial for treating cannabis use disorder. CBD may also have pro-cognitive effects; however, its effect on cognition in people with cannabis use disorder is currently unclear.

**Objectives:**

We aimed to assess whether a 4-week CBD treatment impacted cognitive function. We hypothesised that CBD treatment would improve cognition from baseline to week 4, compared to placebo.

**Methods:**

Cognition was assessed as a secondary outcome in a phase 2a randomised, double-blind, parallel-group and placebo-controlled clinical trial of 4-week daily 200 mg, 400 mg and 800 mg CBD for the treatment of cannabis use disorder. Participants had moderate or severe DSM-5 cannabis use disorder and intended to quit cannabis use. Our pre-registered primary cognitive outcome was delayed prose recall. Secondary cognitive outcomes were immediate prose recall, stop signal reaction time, trail-making task performance, verbal fluency and digit span.

**Results:**

Seventy participants were randomly assigned to placebo (*n* = 23), 400 mg CBD (*n* = 24) and 800 mg CBD (*n* = 23). A 200 mg group was eliminated from the trial because it was an inefficacious dose at interim analysis (*n* = 12) and was not analysed here. For the primary cognitive outcome, there was no effect of CBD compared to placebo, evidenced by a lack of dose-by-time interaction at 400 mg (0.46, 95%CIs: − 1.41, 2.54) and 800 mg (0.89, 95%CIs: − 0.99, 2.81). There was no effect of CBD compared to placebo on secondary cognitive outcomes, except backwards digit span which increased following 800 mg CBD (0.30, 95%CIs: 0.02, 0.58).

**Conclusions:**

In this clinical trial for cannabis use disorder, CBD did not influence delayed verbal memory. CBD did not have broad cognitive effects but 800 mg daily treatment may improve working memory manipulation.

**Clinical trial registration:**

The trial was registered with ClinicalTrials.gov (NCT02044809) and the EU Clinical Trials Register (2013–000,361-36).

**Supplementary Information:**

The online version contains supplementary material available at 10.1007/s00213-022-06303-5.

## Introduction

Cannabis use disorder (CUD), a pattern of cannabis use causing significant impairment or distress, affects an estimated 22 million individuals worldwide (Degenhardt et al. 2018). Cannabis use is responsible for a rising number of new treatment entrants to drug services in almost every world region (United Nations 2020). Psychosocial treatment options are available for CUD; however, these show only modest efficacy, and outcomes in the long term are unclear (Lees et al. 2021). Furthermore, there is currently no approved pharmacotherapy for the treatment of CUD.

Chronic cannabis use is associated with impairment in cognitive function, particularly verbal learning and memory (Broyd et al. 2016; H. V. Curran et al. 2016; Lovell et al. 2020; Zhornitsky et al. 2021). Such use could impact upon other cognitive functions including response inhibition, working memory and verbal fluency, though the evidence is mixed (Broyd et al. 2016). These cognitive impairments may be related to residual effects of cannabis exposure, though evidence for the impact of abstinence on recovery of cognitive function is mixed (Lovell et al. 2020). Studies suggest that people attending treatment for cannabis problems may have impaired cognition (Aharonovich et al. 2018; Bruijnen et al. 2019; Solowij et al. 2002). Deficits in cognition may have a detrimental impact on daily functioning in people who use cannabis. Therefore, if a potential treatment for CUD had pro-cognitive effects, this could be of major benefit to people seeking treatment for CUD. Most pharmacological treatment trials for CUD have not assessed changes in cognitive function and their cognitive effects are unknown. A trial of N-acetylcysteine for CUD found those who reduced their cannabis use (either consistent or recent abstinence; across both treatment and placebo groups), performed significantly better on memory and psychomotor scores compared to those who continued use (Roten et al. 2015). A trial of gabapentin for CUD found a general improvement in cognitive function in the treatment group compared to the placebo; however, this trial had a notably high dropout rate (Mason et al. 2012). Conversely, some pharmacotherapies (such as formulations containing delta-9-tetrahydrocannabinol (THC) or benzodiazepines) could have a detrimental effect on cognition. Cognition therefore represents an important outcome for pharmacological treatments for CUD.

Cannabidiol (CBD) a constituent of cannabis, shows potential as a treatment for CUD (T. P. Freeman et al. 2020; Prud’homme et al. 2015) and is thought to have pro-cognitive effects. Some studies have indicated that CBD may reduce the detrimental effect of THC on cognitive function, though results are mixed (A. M. Freeman et al. 2019). For example, pre-treatment with 600 mg oral CBD reduced the impairing effect of 1.5 mg IV THC on a delayed verbal memory task, compared to pre-treatment with a placebo (Englund et al. 2013). However, another study administering vaporised cannabinoids (C. J. A. Morgan et al. 2018) found that a small dose (16 mg) of CBD did not influence the effects of 8 mg THC on a verbal learning and memory task. Furthermore, the 16 mg CBD dose alone did not significantly affect task performance compared to the placebo.

Naturalistic studies indicate that the level of CBD in the cannabis a person uses may affect verbal learning and memory performance. One study assessed prose recall performance when participants were intoxicated with their own cannabis (C. J. A. Morgan et al. 2010). Those who used cannabis with lower levels of CBD showed poorer performance on immediate and delayed recall when intoxicated compared to those who used cannabis with higher levels of CBD. There was no difference in concentrations of THC between the two groups, and they showed the same level of memory performance when they were not intoxicated. A study in Colorado found that verbal recognition accuracy decreased after acute use of high-THC strains of cannabis, whereas there was no difference in task performance after strains containing both THC and CBD (T. Curran et al. 2020). However, the combined THC and CBD strain group contained significantly less THC than the THC-only strain group; therefore, this improvement could have been due to decreased THC levels rather than the presence of CBD.

Clinical trial data provide preliminary evidence that CBD may benefit cognitive performance. A randomised trial in healthy participants of single-dose vaporised CBD e-liquid (12.5 mg CBD) found better verbal episodic memory performance (but not attention or working memory) after acute CBD administration compared to placebo (Hotz et al. 2021). A trial of 6-week oral daily 1000 mg CBD treatment in people with psychosis found an improvement in the motor speed domain of a cognitive test battery compared to a placebo (McGuire et al. 2018). However, oral daily 600 mg CBD for the treatment of schizophrenia did not increase performance on a composite measure of cognition or on a verbal learning and memory task after a 6-week treatment vs placebo (Boggs et al. 2018). Finally, compared to baseline, performance on a verbal learning task and a measure of attentional switching was significantly improved at the end of 10-week, open-label daily 200 mg oral CBD treatment (Solowij et al. 2018). However, this study lacked a placebo control group. Taken together, the evidence suggests that CBD may impact cognitive performance, though this may depend on the facet of cognition measured, the sample employed and the dose and method of administration. No previous studies have investigated the effects of CBD on cognition in CUD, and there is clearly a need for high-quality, placebo-controlled trials.

Here, we present data on secondary cognitive outcomes from a randomised, phase 2a, double-blind and parallel-group clinical trial of CBD for the treatment of CUD (primary outcomes on cannabis use from this trial have previously been reported (T. P. Freeman et al. 2020)). The following cognitive outcomes were included: prose recall immediate and delayed, stop signal reaction time, trail-making task (Part A, Part B–A), digit span (forwards and backwards) and verbal fluency (letter, semantic and drug). Hypotheses for this analysis as well as the outcomes used for each task were preregistered on the Open Science Framework prior to analysis (https://osf.io/xdjha/). We hypothesised that CBD treatment would improve performance on all tasks from baseline to week 4, compared to placebo. Based on previous evidence (Englund et al. 2013; C. J. A. Morgan et al. 2010), verbal memory performance as measured using the delayed measurement of the prose recall task was chosen as the primary outcome, with all other measures treated as secondary outcomes.

## Method

### Participants

Participants were recruited through website advertisements, forums and through flyers in the local community. They met the following inclusion criteria: aged 16–60, CUD of at least moderate severity (≥ 4 symptoms, assessed by clinical interview for DSM-5 symptoms, conducted by trained psychologists), capacity to give written informed consent, expressed a desire and intention to stop using cannabis within the upcoming month, had one or more unsuccessful prior attempts to quit their cannabis use, co-administered cannabis with tobacco, provided a positive urine sample for THC-COOH and for women, provided a negative pregnancy test within the 7 days prior to starting treatment. Women of childbearing potential and all men were required to use an effective method of contraception (oral, injected, implemented, barrier or true abstinence), from the time of consent until 6 weeks after the end of treatment. Initial criteria for participants to be aged 16–26, with vital signs within normal limits were removed early in the trial to increase the generalisability of findings. Exclusion criteria included the following: (1) current pregnancy or breastfeeding, (2) allergies to CBD, microcrystalline cellulose or gelatine, (3) prescribed psychotropic drug use, (4) use of illicit drugs (other than cannabis) 2 or more times per month at screening, (5) inaccurate self-reported drug use confirmed by a positive urine test for drugs that were not reported during screening, (6) current or previous self-reported diagnosis of a psychotic disorder, (7) physical health problem deemed clinically significant and (8) not speaking English.

### Procedures and measures

The trial was approved by the UK Health Research Authority (13/EE/0303) and the UK Medicines and Healthcare Regulatory Agency (20,363/0325/001–0001) and was prospectively registered with ClinicalTrials.gov (NCT02044809) and the EU Clinical Trials Register (2013–000,361-36). The trial was a single-centre, randomised, double-blind, placebo-controlled and parallel-group study conducted at the Clinical Psychopharmacology Unit, UCL, in central London from May 2014–June 2017. Due to a lack of funding, a subsequent phase 2b stage trial that had been planned was not initiated, and the trial ended in May 2018. The trial protocol can be found at https://osf.io/3cbef/.

After an initial telephone screening, participants attended an in-person screening visit to determine their eligibility prior to randomisation. The trial statistician (GB) generated the randomisation sequence using block randomisation, with a block size equivalent to the number of treatment groups in the randomisation code. The randomisation code was held by the emergency unblinding service (Sealed Envelope, London, UK) and the drug manufacturer for labelling before shipping to the trial site. Researchers and participants remained masked for the duration of the trial. Only masked investigators enrolled participants, assigned participants to interventions, did assessments and entered data. Unmasking occurred after the database had been locked by the trial statistician.

Synthetic, laboratory-synthesised CBD was obtained from STI Pharmaceuticals (Brentwood, UK) and manufactured by Nova Laboratories (Leicester, UK). The first treatment stage of the trial involved twice daily at-home ingestion of two gelatine capsules containing microcrystalline cellulose filler and CBD in total doses of 200 mg, 400 mg, 800 mg or 0 mg (placebo) for 4 weeks. Capsules were identical in size and participants were instructed to take each of the two doses 12 h apart. Text reminders were sent to participants at these pre-arranged times to improve compliance. Instructions were not given for taking the doses with/without food. Participants’ adherence to the treatment schedule was monitored via the return of dosette boxes and self-report of use using diary cards.

The trial was conducted to determine the most effective dose of CBD in reducing cannabis use and consisted of two stages. In the first stage, *n* = 12 participants were recruited to each of the four treatment groups (1:1:1:1). Once these participants completed the 4-week treatment, a planned interim analysis using Bayesian models computed the likelihood that each CBD dose was the most effective dose according to the primary endpoints (urinary THC-COOH/creatinine and days with abstinence from cannabis). This interim analysis determined that 200 mg CBD was inefficacous, and so this dose was eliminated from the trial with no further randomisation to this group. The second stage of the trial involved further participants being randomised to expand the 400 mg, 800 mg and placebo groups (1:1:1), up to a sample size of *n* = 24 (400 mg CBD), *n* = 23 (800 mg CBD) and *n* = 23 (placebo). The final analysis of the primary endpoints indicated that both 400 mg CBD and 800 mg CBD were more efficacious than placebo for reducing cannabis use. In line with the analysis of the primary endpoint in the main trial, this secondary analysis of cognitive outcomes analysed data from the final sample size of *n* = 24 (400 mg CBD), *n* = 23 (800 mg CBD) and *n* = 23 (placebo). As the 200 mg group had a smaller sample size of 12 participants, it was not included in this secondary analysis of cognitive outcomes to maximise statistical power.

Participants attended site visits once weekly during treatment. All participants received six 30-min sessions of motivational interviewing (Miller & Rollnick 2012) at screening, baseline and weeks 1–4 of treatment, delivered by trained psychologists. During the first session, a quit date was planned to coincide with the baseline visit. Participants who did not self-determine a suitable target quit date in our trial were not eligible to be randomised (as per our eligibility criteria). However, they were given the opportunity to set a target quit date in the future if their situation changed, as long as they had not previously been randomised. Participants were not offered active treatment after participation in the trial. Cognitive tasks as well as assessments of cannabis use (urine sample and timeline follow-back interview) were completed at the baseline visit, week 4 (end of treatment) and week 12 (follow-up). Urine samples were collected using temperature-monitored cups (Galle pot, Synergy Health, Abergavenny, UK) to ensure adherence. Samples were stored in 10 mL polypropylene tubes at − 80 °C before analysis using liquid chromatography-tandem mass spectrometry by ABS Laboratories (Hertford, UK) with a lower limit of THC-COOH quantification of 1 ng/mL.

### Cognitive outcomes

#### Prose recall

Verbal episodic memory performance was assessed using the prose recall task, a modified measurement from the Rivermead Behavioural Memory Test battery (Wilson et al. 1989). A 30-s clip of a news report was played through headphones. Participants were instructed to write down as much as they could remember from the audio clip once it had finished (*immediate* recall). After an interference delay of 30 min, they were asked again to write down as much as they could remember (*delayed* recall). The number of ‘idea units’ out of 21 was recorded at both time points.

#### Stop signal

Response inhibition was assessed using the stop signal task. During this computer-based task, white arrows appeared sequentially in the centre of the screen. Participants pressed a key on the keyboard based on the direction that the arrow was pointing in (left or right). In 25% of trials, the arrow turned from white to blue, following a variable delay. In these trials, participants were instructed to not press any arrow key, thereby inhibiting their initiated response. There was one block of 32 practice trials and three blocks of 64 experimental trials. Staircase tracking ensured that the delay occurred such that the participant had approximately a 50% chance of successfully inhibiting their response. The outcome (stop signal reaction time; SSRT) was generated via a computer programme ‘STOP-IT’ (Verbruggen et al. 2008).

#### Trail-making task

Psychomotor speed, attention and task switching were assessed using the trail-making task (TMT; Reitan 1986). In Part A, participants were asked to draw a line using a pencil to connect consecutive numbers on paper. In Part B, they were required to draw a line to connect numbers and letters on paper alternatively in numerical and alphabetical order (e.g. A-1-B-2-C-3). The length of time taken to complete each part of the task was recorded. The completion time for Part A was subtracted from Part B to obtain a measure of task switching adjusted for psychomotor speed.

#### Digit span

Working memory was assessed using the digit span task (Wechsler 1997). The researcher read a series of digit strings to the participant who was then asked to verbally recall the digits in the same order in which they appeared (forwards), or in the opposite order (backwards). The number of items increased every two strings, and the longest string correctly recalled was recorded. The maximum score was 12 for both conditions.

#### Verbal fluency

Finally, verbal fluency was assessed using a letter (phonemic), category (semantic) and drug (cannabis) fluency prompts. Participants were required to generate as many words related to each prompt as they could within 1 min. The number of relevant, unique words mentioned was recorded and summed for each variation.

Alternate versions of all cognitive tasks were used at each assessment, with the exception of the stop signal task which used a randomised trial design.

#### Statistical analysis

A power analysis conducted for the primary outcome of the main trial (time by group interaction on reduction in cannabis use) indicated that 12 participants per group would provide 80% power to detect an effect of CBD on cannabis use, based on a previous study of CBD on cigarette use in tobacco smokers (T. P. Freeman et al. 2020; C. J. Morgan et al. 2013). However, as the previous study was conducted in a different population and the effect size in this context was unknown, an interim analysis was planned at *n* = 12 per treatment group and group size was capped at a maximum of *n* = 24 per treatment group.

The effect of CBD treatment compared to placebo on each cognitive outcome was analysed using linear mixed-effects models, using the “lme4” package in R. Data from all patients randomly assigned to placebo, 400 mg CBD and 800 mg CBD groups were analysed on an intention-to-treat basis. Models included data from baseline and end of treatment only (week 4) in order to focus on the effect of the treatment period, consistent with the primary endpoint analysis of the main trial (T. P. Freeman et al. 2020), to minimise risk of missing data and increase statistical power. Prior to analysing the data, we pre-registered the primary outcome for this analysis of cognitive assessments as the delayed measurement of the prose recall task (https://osf.io/xdjha/). All other cognitive outcomes were treated as secondary outcomes. Models included fixed effects of dose (placebo, 400 mg, 800 mg CBD), time (baseline, week 4) and dose-by-time interaction. The effect of dose and time was incremental with respect to the reference categories (‘placebo’ and ‘baseline’ respectively). Participant was added to the model as a random intercept. In all cases, the random effect improved model fit/accounted for greater variance and therefore was retained in all models. Planned contrasts of change in performance from baseline to week 4 were assessed and stratified across treatment groups. Bootstrapped 95% confidence intervals were used as inference criteria. Analyses were conducted in R version 4.1.1.

## Results

**Fig. 1:**
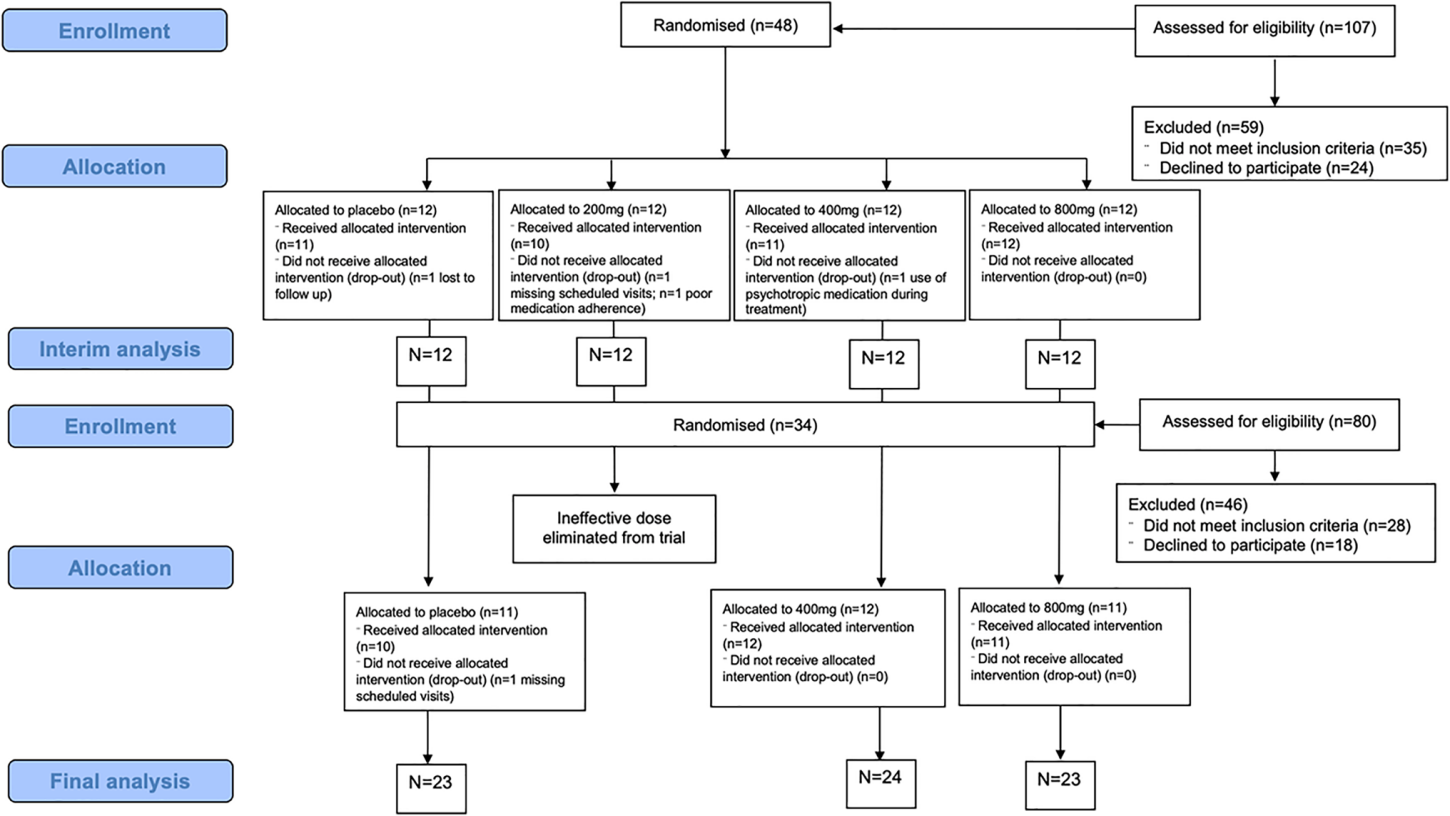
CONSORT flow diagram

Demographic details of participants in each treatment group are provided in Table 1. The sample (*n* = 70) consisted primarily of young adult males (mean age = 26.3 years, % male = 71.43). Three participants did not receive their full allocated intervention (Placebo *n* = 2, 400 mg CBD *n* = 1) due to either missing scheduled visits or the use of psychotropic medication during the treatment period (Fig. 1). However, the attendance rate at the end of treatment visit was very high with only one participant in the placebo group who did not attend (*n* = 69). Of the 70 participants in this study, 67 (96%) adhered to their treatment (evidenced by self-report and returned medication) and attended all treatment week visits within 2 days of the scheduled appointment. Only one participant (400 mg group) who attended the end of treatment visit did not complete the cognitive assessments; therefore, baseline and week 4 data on cognition were available for 97% of participants (*n* = 68). The number of mild and moderate adverse events did not differ between placebo and either CBD doses. No severe adverse events were recorded.

**Table 1:** Baseline participant demographics. Data shown are frequencies and means (standard deviations) as appropriate

	Placebo	400 mg CBD	800 mg CBD
*n*	23	24	23
Age (years)	24.87 (7.44)	26.58 (6.79)	27.43 (5.83)
Gender (M/F)	17/6	17/7	16/7
CUD symptoms	8.61 (1.73)	9.00 (1.25)	8.48 (1.93)
Urinary THC:COOH	343.09 (357.70)	521.02 (484.23)	315.38 (382.28)
Days abstinent from cannabis per week	1.17 (1.61)	0.79 (1.06)	1.65 (2.25)

Days of cannabis used assessed via timeline follow-back interview. CUD symptoms assessed using DSM-5 interview

### Primary outcome: delayed prose recall

**Fig. 2:**
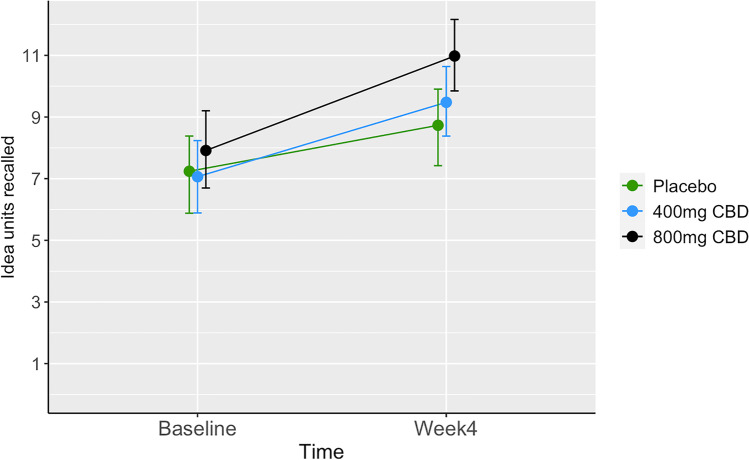
Group means of idea units recalled on the delayed prose recall task, by treatment group at baseline and week 4. Error bars represent bootstrapped 95% confidence intervals

There was no significant dose-by-time interaction on delayed prose recall scores (Table 2). There was a significant main effect of time, indicating improved recall across groups at week 4 compared to baseline. The change in performance stratified by treatment group is displayed in Table 3 and Fig. 2.

**Table 2:** Data show model estimates of the effect of dose, time, and dose by time interaction on delayed prose recall

	Estimate	SE	Df	*t*-value	Lower CI	Upper CI
Intercept	6.11	0.66	111.76	9.29	4.67	7.29
Placebo						
400 mg CBD	0.41	0.92	111.76	0.45	-1.40	2.41
800 mg CBD	1.15	0.93	111.76	1.24	-0.67	3.03
Baseline						
Week 4	1.83	0.71	67.03	2.57	0.45	3.14
Dose by time (placebo, baseline)						
Dose by time (400 mg CBD, week 4)	0.46	1.00	67.00	0.46	-1.41	2.54
Dose by time (800 mg CBD, week 4)	0.89	1.00	66.44	0.89	-0.99	2.81

*CI*, 95% confidence interval, bootstrapped. *SE*, standard error. *Df*, degrees of freedom

**Table 3:** Change from baseline to week 4 (end of treatment) for scores on prose recall delayed, by treatment group

Contrast	Treatment	Estimate	SE	Df	Lower CI	Upper CI
Week 4 — baseline	Placebo	0.91	0.36	66.34	0.22	1.57
Week 4 — baseline	400 mg CBD	1.14	0.35	66.29	0.48	1.86
Week 4 — baseline	800 mg CBD	1.36	0.35	65.16	0.65	2.05

*CI*, 95% confidence interval, bootstrapped. *SE*, standard error. *Df*, degrees of freedom

### Secondary outcomes

**Fig. 3:**
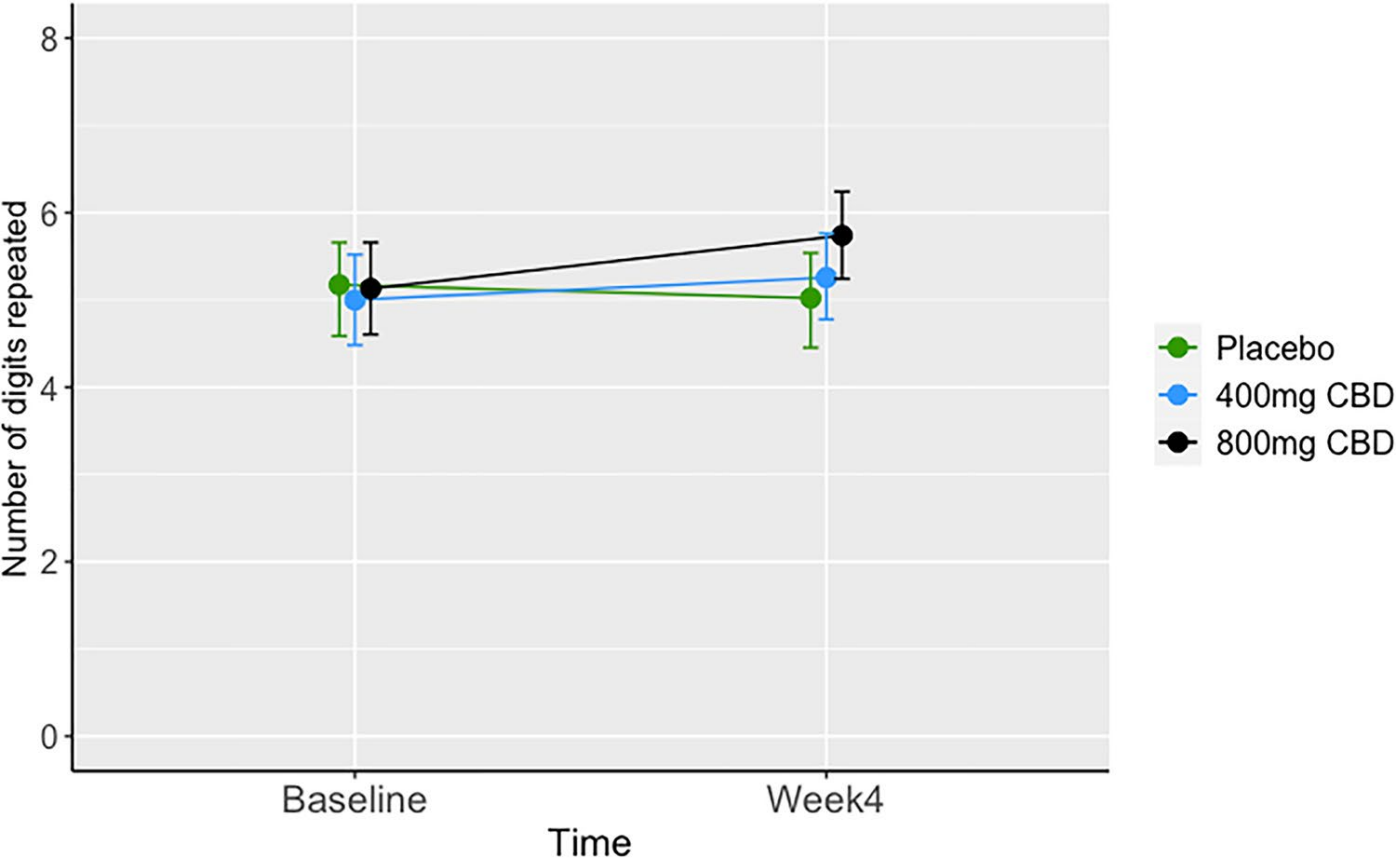
Group means of number of digits recall backwards, by treatment group at baseline and week 4. Error bars represent bootstrapped 95% confidence intervals

For the backwards digit span, there was a significant dose-by-time interaction at 800 mg CBD (0.76, 95%CIs: 0.01, 1.54), but not at 400 mg CBD (0.41, 95%CIs: − 0.34, 1.25). The change in performance was 0.30 (95%CIs: 0.02, 0.58) in the 800 mg group; 0.13, (95%CIs: − 0.14, 0.42) in the 400 mg group, and − 0.08 (95%CIs: − 0.35, 0.1 in the placebo group; see Fig. 3). There was no main effect of CBD dose or time on backwards digit span.

For all other secondary outcomes (SSRT, TMT A, TMT B-A, forwards digit span, letter fluency, category fluency and drug fluency), there was no significant dose-by-time interaction at 400 mg or 800 mg CBD. There was also no main effect of dose for any secondary outcome.

For immediate prose recall, TMT A and category fluency, there was a significant main effect of time. See supplementary materials for details of all analyses of secondary outcomes.

### Exploratory analyses

To determine if there was an effect of change in cannabis use on cognitive function, we added urinary THC:COOH levels (measured at both baseline and week 4) as a time-varying covariate and an interaction term of urinary THC:COOH by time as fixed effects to the models. For all models, adding these as fixed effects did not influence the main results, with no evidence for the effect of CBD compared to placebo on cognitive outcomes. The dose-by-time interaction at 800 mg for backwards digit span remained significant after adjustment (0.81, 95%CIs: 0.07–1.56). For prose recall delayed and immediate, as well as category fluency, the effect of time remained significant after adjustment for urinary THC:COOH. Time was no longer significant in the model for psychomotor speed (TMT Part A) after adjustment.

## Discussion

We used a comprehensive cognitive task battery to assess performance before and after 4-week treatment with daily oral 400 mg CBD, 800 mg CBD or placebo in a double-blind, randomised and placebo-controlled clinical trial. Contrary to our hypotheses, there was no effect of CBD on delayed prose recall compared to the placebo. There was a lack of effects of CBD on other cognitive outcomes, apart from a significant dose-by-time interaction indicating that 800 mg CBD improved performance from baseline to week 4 for backwards digit span, a measure of working memory. On the delayed prose recall task (the pre-registered primary outcome), performance increased in all groups from baseline to week 4. Taken together, these results suggest that CBD may not produce broad cognitive effects in people with CUD but could benefit working memory manipulation.

Previous evidence has indicated that verbal memory is the key cognitive domain impacted by CBD treatment; however, this was not supported by the current findings. An open-label trial of 200 mg daily CBD over 10 weeks found better verbal learning and memory performance at end of treatment compared to baseline (Solowij et al. 2018); however, that trial lacked a placebo group so this effect could reflect practice on the task. A single-dose placebo-controlled trial of 12 mg CBD e-liquid improved verbal memory performance compared to placebo (Hotz et al. 2021). The sample size of both trials was small (*n* = 20; *n* = 34), and the effect size for the difference in performance was also small across both studies (0.53, 0.028). This trial had a larger sample size than these two previous trials and included a placebo control as well as a dose–response design.

We found that 800 mg CBD improved the manipulation of information in working memory indexed via backwards digit span and did not affect maintenance indexed by forward digit span. Previous studies have indicated null effects of CBD on digit span tasks. One experimental study administering a single dose of 600 mg oral CBD did not find a significant effect on forwards or backwards digit span compared to a placebo (Bloomfield et al. 2020). Another study found no significant effect of CBD treatment compared to placebo on backwards digit span performance after 1.5 mg IV THC (Englund et al. 2013). The current findings indicate that CBD treatment may impact on working memory when given daily at 800 mg. However, backwards digit span was one of several secondary outcomes in this analysis, and therefore, this result should be considered preliminary until replicated. At the same time, these findings highlight working memory as a focus for future hypothesis-driven studies of the cognitive effects of CBD.

Of note, performance improved across some cognitive outcomes from baseline to end of treatment, including both measurements of the prose recall task, psychomotor speed, and category fluency. There are several potential explanations for this. Firstly, cognition may have improved due to lower exposure to THC or general improvement in wellbeing caused by the reduction in CUD over the trial. However, exploratory analyses indicated that the effect of time remained significant after adjustment for urinary THC:COOH levels (except for psychomotor speed), indicating that this was not responsible for the increase in performance. Across the trial, all groups including the placebo group reduced their cannabis use considerably, which might potentially explain why the addition of a urinary marker of recent cannabis use did not alter the pattern of results. Moreover, there was no evidence for the effect of CBD on cognition being greater than placebo in the adjusted models. Secondly, the cognitive tasks used might be sensitive to practice effects, with participants scoring higher at the end of treatment as they are more familiar with the task and its instructions. It is also possible that there was an exposure effect to the testing environment over time which may reduce participants’ anxiety.

This analysis benefits from robust RCT methodology including randomisation, double-blinding and placebo control. The trial used an intention-to-treat analysis, with 97% of participants providing data at baseline and end of treatment. The 4-week exposure period and two doses of CBD investigated allow for a thorough investigation of daily CBD treatment on cognition, using pre-registered hypotheses. One limitation is that the trial may have had limited power to detect potentially true effects of CBD with small effect sizes. As this was an analysis of a fixed sample from an existing dataset, an a priori power analysis could not be conducted. Given the 1.5 times larger increase for delayed prose recall and 2 times larger increase for immediate prose recall in the 800 mg group compared to placebo, there may be potential for pro-cognitive effects of daily CBD treatment on verbal memory, but larger sample sizes would be needed to detect small effect sizes. Another consideration to note is that all groups (including placebo) received motivational interviewing. This technique has demonstrated efficacy in reducing cannabis use in previous trials (Lees et al. 2021); however, whether motivational interviewing improves cognition in people with CUD has not been assessed. It will therefore be valuable to assess the impact of CBD against a placebo-only control group with no concomitant psychological treatment in future research.

In conclusion, this randomised, double blind and placebo-controlled trial found that 400 mg and 800 mg of CBD treatment did not significantly improve verbal learning and memory performance over 4 weeks, compared to placebo. There was evidence of a small beneficial effect of CBD on working memory as assessed by the backwards digit span.

## Supplementary Information

Below is the link to the electronic supplementary material.

Supplementary file1 (PDF 429 KB)

Supplementary file2 (PDF 264 KB)

## Data Availability

The participants of this study did not give written consent for their data to be shared publicly, so the research supporting data is not available.
