# Supplementary material for: Effect of four-week cannabidiol treatment on cognitive function: secondary outcomes from a randomised clinical trial for the treatment of cannabis use disorder
Source: Psychopharmacology (Berl). 2023 Jan 4;240(2):337–46. doi: 10.1007/s00213-022-06303-5 (PMC9879826; doi:10.1007/s00213-022-06303-5)
Supplement: Supplementary file 1 — (PDF 429 KB) [file 213_2022_6303_MOESM1_ESM.pdf]

Effect of four-week cannabidiol treatment on cognitive function: secondary outcomes from a randomised clinical trial for the treatment of cannabis use disorder

Psychopharmacology

Authors
Rachel Lees, Lindsey Hines, Chandni Hindocha, Gianluca Baio, Natacha D C Shaban, George Stothart, Ali Mofeez, Celia J A Morgan, H Valerie Curran, Tom P Freeman

Corresponding author: Rachel Lees (rhl32@bath.ac.uk)

SUPPLEMENTARY MATERIALS

Table of Contents 1
Secondary outcome analyses 2
Immediate prose recall 2
SSRT 4
Trail making task – Part A 6
Trail making task – Part B- Part A 7
Digit Span 9
Backwards digit span 10
Letter (phonemic) fluency 12
Category (semantic) fluency 14
Drug Fluency 16
Exploratory Bayes Factors 18

Secondary outcome analyses

Immediate prose recall

Table S1
Data show model estimates of the effect of dose, time, and dose by time interaction on immediate prose recall.

	Estimate	SE	Df	*t*-value	Lower CI	Upper CI
Intercept	7.24	0.62	117.48	11.63	5.88	8.38
Placebo	-	-	-	-	-	-
400mg CBD	-0.18	0.87	117.48	-0.20	-1.90	1.72
800mg CBD	0.67	0.88	117.48	0.77	-1.02	2.47
Baseline	-	-	-	-	-	-
Week 4	1.49	0.72	67.34	2.09	0.11	2.83
Dose by time (Placebo, Baseline)	-	-	-	-	-	-
Dose by time (400mg CBD, Week 4)	0.92	1.00	67.32	0.92	-0.95	3.02
Dose by time (800mg CBD, Week 4)	1.57	1.00	66.71	1.57	-0.31	3.50

CI = 95% confidence interval, bootstrapped. SE = Standard Error. Df = Degrees of freedom.

Table S2
Change from baseline to week 4 (end of treatment) for scores on immediate prose recall, by treatment group.

Contrast	Treatment	Estimate	SE	Df	Lower CI	Upper CI
Week 4 - Baseline	Placebo	0.75	0.36	66.47	0.06	1.41
Week 4 - Baseline	400mg CBD	1.21	0.35	66.42	0.53	1.92
Week 4 - Baseline	800mg CBD	1.53	0.35	65.20	0.82	2.23

CI = 95% confidence interval, bootstrapped. SE = Standard Error. Df = Degrees of freedom.

Figure S1
Group means of idea units recalled at the immediate measurement of the prose recall task, by treatment group at baseline and week 4. Error bars represent bootstrapped 95% confidence intervals.

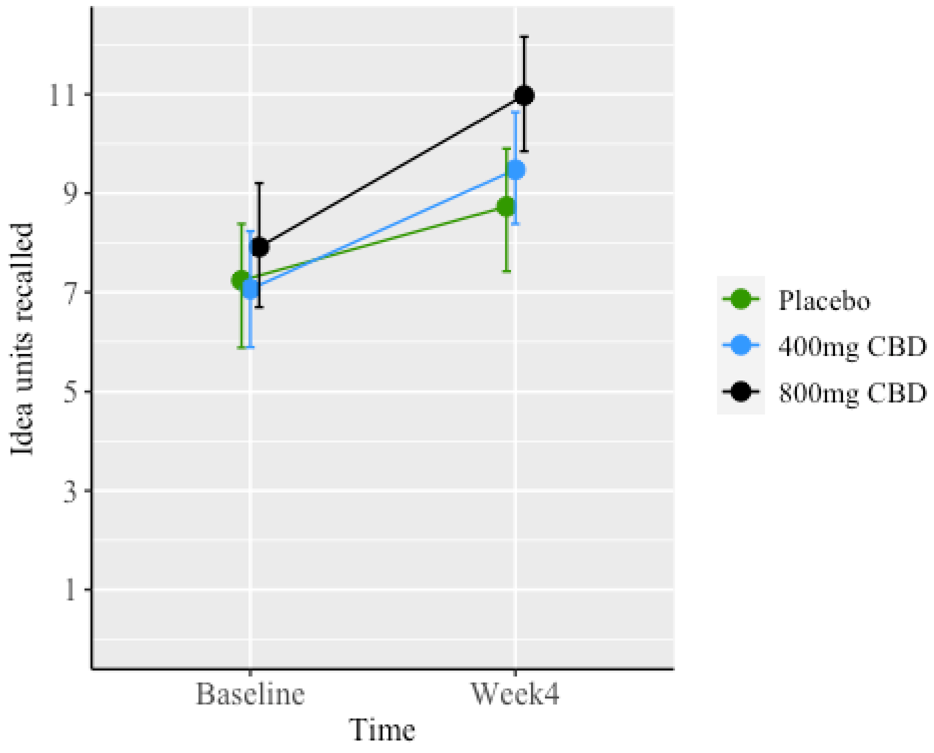

SSRT

Table S3

Data show model estimates of the effect of dose, time, and dose by time interaction on SSRT.

	Estimate	SE	Df	*t*-value	Lower CI	Upper CI
Intercept	257.21	11.19	110.26	22.99	232.78	277.34
Placebo	-	-	-	-	-	-
400mg CBD	-5.50	15.65	110.26	-0.35	-36.47	28.58
800mg CBD	-3.27	15.82	110.26	-0.21	-34.22	28.40
Baseline	-	-	-	-	-	-
Week 4	-18.44	11.90	66.96	-1.55	-41.56	3.52
Dose by time (Placebo, Baseline)	-	-	-	-	-	-
Dose by time (400mg CBD, Week 4)	7.36	16.64	66.94	0.44	-23.80	42.22
Dose by time (800mg CBD, Week 4)	5.01	16.69	66.39	0.30	-26.41	37.22

CI = 95% confidence interval, bootstrapped. SE = Standard Error. Df = Degrees of freedom.

Table S4

Change from baseline to week 4 (end of treatment) for scores on SSRT, by treatment group.

Contrast	Treatment	Estimate	SE	Df	Lower CI	Upper CI
Week 4 - Baseline	Placebo	-9.22	5.95	66.30	-20.78	1.76
Week 4 - Baseline	400mg CBD	-5.54	5.82	66.26	-16.68	6.46
Week 4 - Baseline	800mg CBD	-6.72	5.85	65.15	-18.60	4.87

CI = 95% confidence interval, bootstrapped. SE = Standard Error. Df = Degrees of freedom.

Figure S2
Group means of SSRT score, by treatment group at baseline and week 4. Error bars represent bootstrapped 95% confidence intervals.

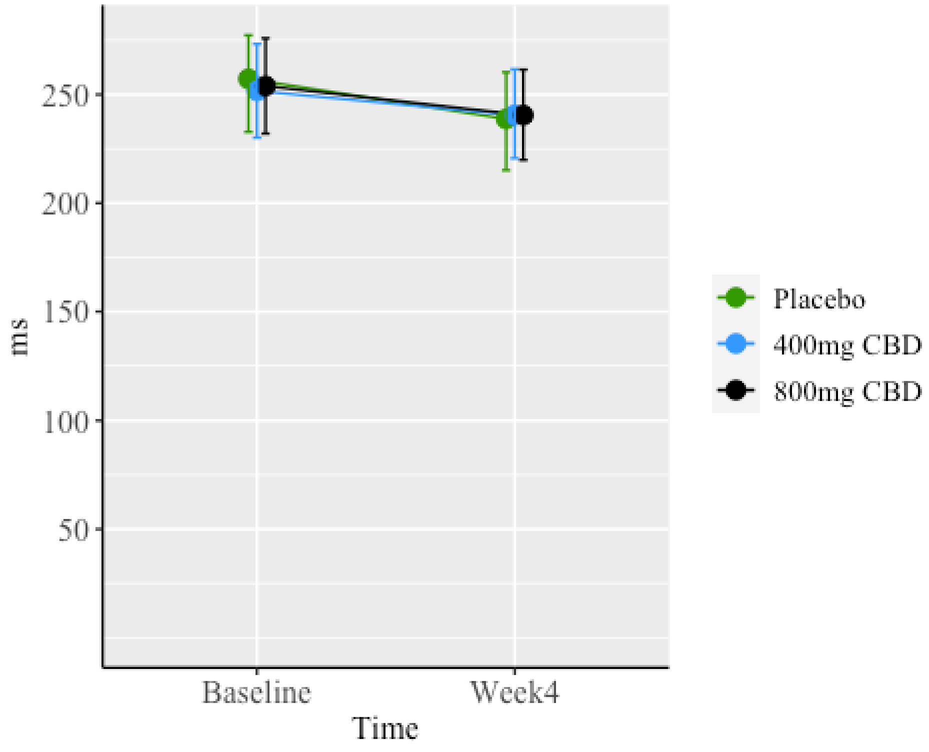

Trail making task – Part A
Table S5
Data show model estimates of the effect of dose, time, and dose by time interaction on Part A of the trail making task

	Estimate	SE	Df	*t*-value	Lower CI	Upper CI
Intercept	24.29	1.43	103.83	16.95	21.15	26.90
Placebo	-	-	-	-	-	-
400mg CBD	1.31	2.01	103.83	0.65	-2.57	5.67
800mg CBD	-2.26	2.03	103.83	-1.12	-6.22	1.80
Baseline	-	-	-	-	-	-
Week 4	-3.51	1.43	65.13	-2.45	-6.30	-0.89
Dose by time (Placebo, Baseline)	-	-	-	-	-	-
Dose by time (400mg CBD, Week 4)	-0.74	2.00	65.10	-0.37	-4.49	3.45
Dose by time (800mg CBD, Week 4)	0.94	2.01	64.60	0.47	-2.83	4.80

CI = 95% confidence interval, bootstrapped. SE = Standard Error. Df = Degrees of freedom.

Table S6
Change from baseline to week 4 (end of treatment) for scores on part A of the trail making task, by treatment group.

Contrast	Treatment	Estimate	SE	Df	Lower CI	Upper CI
Week 4 - Baseline	Placebo	-1.75	0.72	66.18	-3.15	-0.44
Week 4 - Baseline	400mg CBD	-2.13	0.70	66.13	-3.47	-0.67
Week 4 - Baseline	800mg CBD	-1.28	0.70	65.11	-2.71	0.11

CI = 95% confidence interval, bootstrapped. SE = Standard Error. Df = Degrees of freedom.

Trail making task – Part B- Part A

Table S7

Data show model estimates of the effect of dose, time, and dose by time interaction on Part B – Part A of the trail making task

	Estimate	SE	Df	*t*-value	Lower CI	Upper CI
Intercept	36.94	4.77	120.60	7.75	26.79	45.53
Placebo	-	-	-	-	-	-
400mg CBD	-9.61	6.67	120.60	-1.44	-22.54	4.75
800mg CBD	-10.35	6.74	120.60	-1.54	-23.31	3.31
Baseline	-	-	-	-	-	-
Week 4	1.35	5.67	67.25	0.24	-9.63	11.91
Dose by time (Placebo, Baseline)	-	-	-	-	-	-
Dose by time (400mg CBD, Week 4)	0.52	7.93	67.22	0.07	-14.28	17.09
Dose by time (800mg CBD, Week 4)	12.09	7.96	66.60	1.52	-2.82	27.32

CI = 95% confidence interval, bootstrapped. SE = Standard Error. Df = Degrees of freedom.

Table S8

Change from baseline to week 4 (end of treatment) for scores on Part B – Part A of the trail making task, by treatment group.

Contrast	Treatment	Estimate	SE	Df	Lower CI	Upper CI
Week 4 - Baseline	Placebo	0.67	2.84	66.55	-4.82	5.95
Week 4 - Baseline	400mg CBD	0.93	2.77	66.50	-4.44	6.53
Week 4 - Baseline	800mg CBD	6.72	2.79	65.23	1.05	12.25

CI = 95% confidence interval, bootstrapped. SE = Standard Error. Df = Degrees of freedom.

Figure S3
Group means of time taken to complete Part A of the Trail making Task (3a), and Part B – A (3b), by treatment group at baseline and week 4. Error bars represent bootstrapped 95% confidence
intervals.

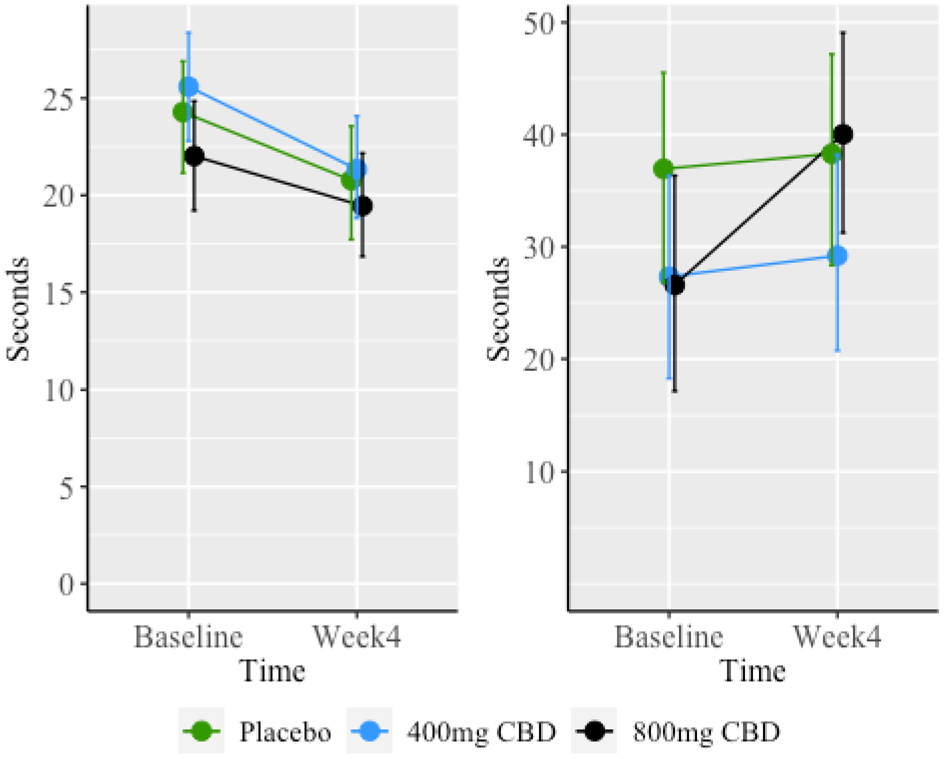

Digit Span

Table S9
Data show model estimates of the effect of dose, time, and dose by time interaction on digit span.

	Estimate	SE	Df	*t*-value	Lower CI	Upper CI
Intercept	6.96	0.26	97.79	27.26	6.40	7.43
Placebo	-	-	-	-	-	-
400mg CBD	0.63	0.36	97.79	1.76	-0.07	1.39
800mg CBD	0.43	0.36	97.79	1.20	-0.27	1.17
Baseline	-	-	-	-	-	-
Week 4	0.18	0.23	66.20	0.78	-0.28	0.61
Dose by time (Placebo, Baseline)	-	-	-	-	-	-
Dose by time (400mg CBD, Week 4)	-0.16	0.33	66.18	-0.50	-0.77	0.52
Dose by time (800mg CBD, Week 4)	0.04	0.33	65.74	0.11	-0.58	0.66

CI = 95% confidence interval, bootstrapped. SE = Standard Error. Df = Degrees of freedom.

Table S10
Change from baseline to week 4 (end of treatment) for digits repeated on the digit span test, by treatment group.

Contrast	Treatment	Estimate	SE	Df	Lower CI	Upper CI
Week 4 - Baseline	Placebo	0.09	0.12	66.00	-0.14	0.30
Week 4 - Baseline	400mg CBD	0.01	0.11	65.97	-0.21	0.25
Week 4 - Baseline	800mg CBD	0.11	0.11	65.08	-0.12	0.33

CI = 95% confidence interval, bootstrapped. SE = Standard Error. Df = Degrees of freedom.

Backwards digit span

Table S11
Data show model estimates of the effect of dose, time, and dose by time interaction on backwards digit span.

	Estimate	SE	Df	*t*-value	Lower CI	Upper CI
Intercept	5.17	0.27	109.54	19.23	4.59	5.66
Placebo	-	-	-	-	-	-
400mg CBD	-0.17	0.38	109.54	-0.46	-0.92	0.65
800mg CBD	-0.04	0.38	109.54	-0.11	-0.79	0.72
Baseline	-	-	-	-	-	-
Week 4	-0.15	0.29	65.75	-0.53	-0.71	0.38
Dose by time (Placebo, Baseline)	-	-	-	-	-	-
Dose by time (400mg CBD, Week 4)	0.41	0.40	65.72	1.02	-0.34	1.25
Dose by time (800mg CBD, Week 4)	0.76	0.40	65.17	1.90	0.01	1.54

CI = 95% confidence interval, bootstrapped. SE = Standard Error. Df = Degrees of freedom.

Table S12
Change from baseline to week 4 (end of treatment) for digits repeated backwards, by treatment group

Contrast	Treatment	Estimate	SE	Df	Lower CI	Upper CI
Week 4 - Baseline	Placebo	-0.08	0.14	66.30	-0.35	0.19
Week 4 - Baseline	400mg CBD	0.13	0.14	66.25	-0.14	0.42
Week 4 - Baseline	800mg CBD	0.30	0.14	65.15	0.02	0.58

CI = 95% confidence interval, bootstrapped. SE = Standard Error. Df = Degrees of freedom.

Figure S4
Group means on forward and backward digit span by treatment group, at baseline and week 4. Error bars represent bootstrapped 95% confidence intervals.

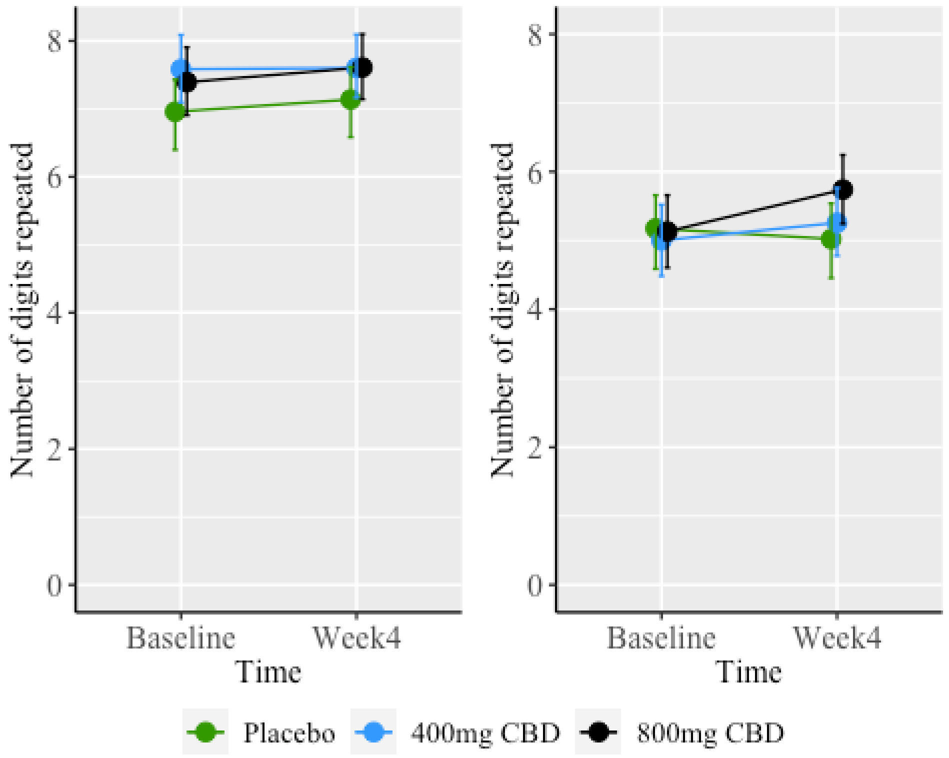

Letter (phonemic) fluency

Table S13
Data show model estimates of the effect of dose, time, and dose by time interaction on letter fluency.

	Estimate	SE	Df	*t*-value	Lower CI	Upper CI
Intercept	12.57	0.99	106.49	12.73	10.40	14.38
Placebo	-	-	-	-	-	
400mg CBD	0.89	1.38	106.49	0.65	-1.82	3.88
800mg CBD	-0.13	1.40	106.49	-0.09	-2.85	2.64
Baseline	-	-	-	-	-	
Week 4	-0.31	1.01	66.61	-0.30	-2.27	1.54
Dose by time (Placebo, Baseline)	-	-	-	-	-	
Dose by time (400mg CBD, Week 4)	-0.88	1.41	66.58	-0.63	-3.52	2.07
Dose by time (800mg CBD, Week 4)	0.13	1.41	66.07	0.09	-2.53	2.85

CI = 95% confidence interval, bootstrapped. SE = Standard Error. Df = Degrees of freedom.

Table S14
Change from baseline to week 4 (end of treatment) for number of letter words recalled, by treatment group.

Contrast	Treatment	Estimate	SE	Df	Lower CI	Upper CI
Week 4 - Baseline	Placebo	-0.15	0.50	66.22	-1.14	0.77
Week 4 - Baseline	400mg CBD	-0.59	0.49	66.17	-1.54	0.43
Week 4 - Baseline	800mg CBD	-0.09	0.50	65.12	-1.09	0.89

CI = 95% confidence interval, bootstrapped. SE = Standard Error. Df = Degrees of freedom.

Figure S5
Group means of number of words recalled on the letter fluency task by treatment group, at baseline and week 4. Error bars represent bootstrapped 95% confidence intervals.

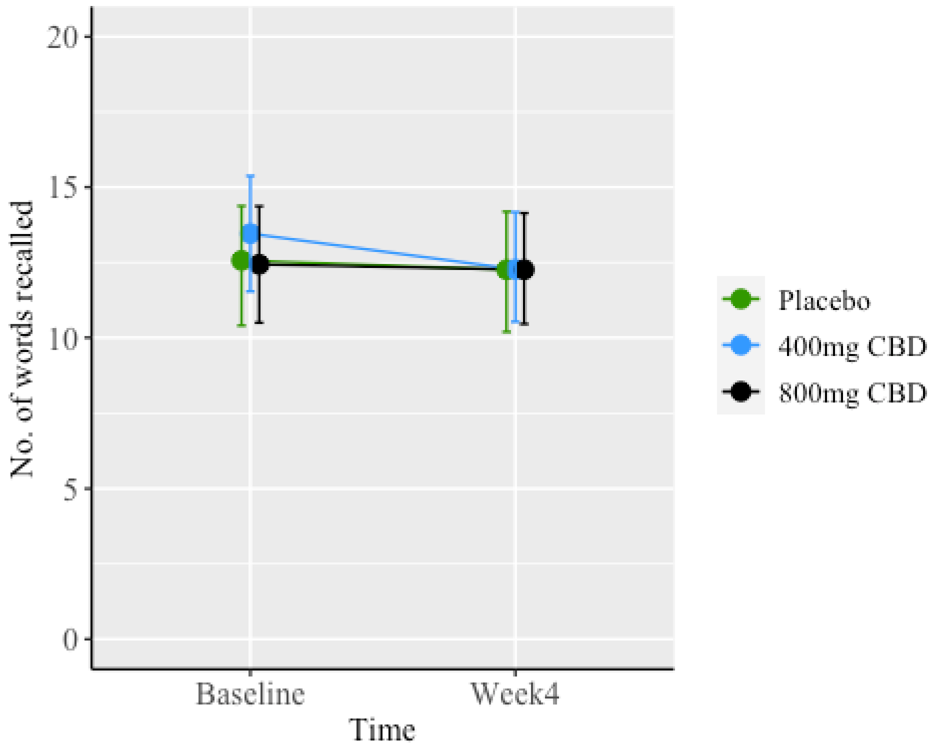

Category (semantic) fluency

Table S15
Data show model estimates of the effect of dose, time, and dose by time interaction on category (semantic) fluency.

	Estimate	SE	Df	*t*-value	Lower CI	Upper CI
Intercept	13.48	0.89	104.00	15.15	11.53	15.10
Placebo	-	-	-	-	-	-
400mg CBD	1.23	1.24	104.00	0.99	-1.16	3.92
800mg CBD	-0.26	1.26	104.00	-0.21	-2.72	2.27
Baseline	-	-	-	-	-	-
Week 4	2.60	0.88	66.61	2.96	0.88	4.21
Dose by time (Placebo, Baseline)	-	-	-	-	-	-
Dose by time (400mg CBD, Week 4)	-1.37	1.23	66.59	-1.11	-3.67	1.21
Dose by time (800mg CBD, Week 4)	-1.34	1.23	66.09	-1.09	-3.66	1.03

CI = 95% confidence interval, bootstrapped. SE = Standard Error. Df = Degrees of freedom.

Table S16
Change from baseline to week 4 (end of treatment) for number of category (semantic) words recalled, by treatment group.

Contrast	Treatment	Estimate	SE	Df	Lower CI	Upper CI
Week 4 - Baseline	Placebo	1.30	0.44	66.16	0.44	2.11
Week 4 - Baseline	400mg CBD	0.62	0.43	66.11	-0.21	1.51
Week 4 - Baseline	800mg CBD	0.63	0.43	65.11	-0.25	1.49

CI = 95% confidence interval, bootstrapped. SE = Standard Error. Df = Degrees of freedom.

Figure S6
Group means of number of words recalled on the category fluency task by treatment group, at baseline and week 4. Error bars represent bootstrapped 95% confidence intervals.

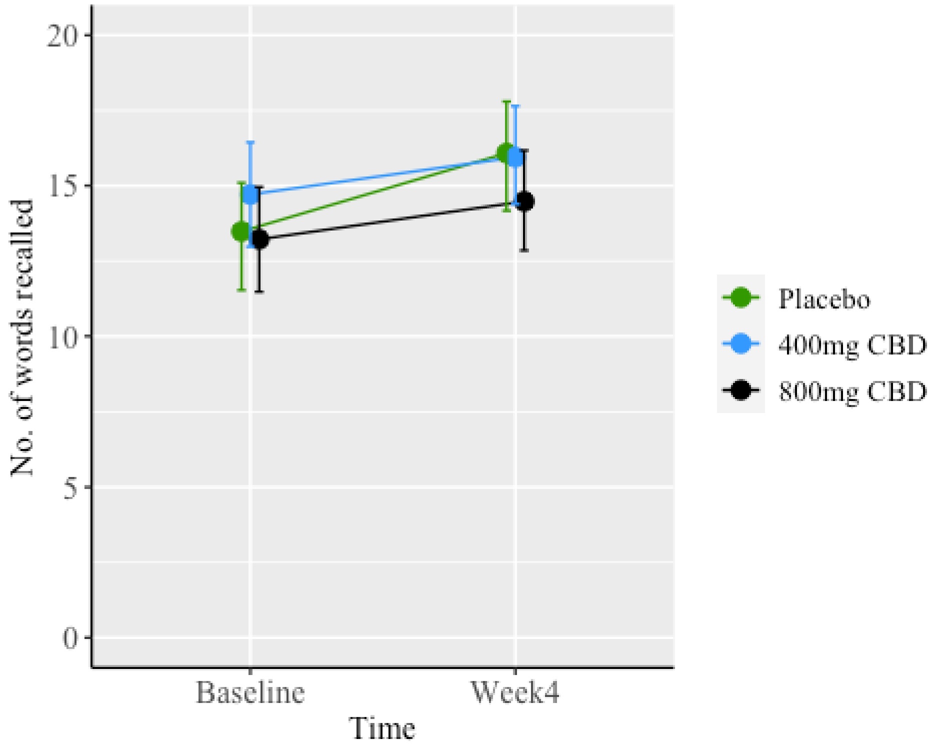

Drug Fluency

Table S17

Data show model estimates of the effect of dose, time, and dose by time interaction on drug (cannabis) fluency.

	Estimate	SE	Df	*t*-value	Lower CI	Upper CI
Intercept	18.78	1.14	88.96	16.51	16.36	20.91
Placebo	-	-	-	-	-	-
400mg CBD	0.68	1.59	88.96	0.42	-2.53	4.08
800mg CBD	-0.43	1.61	88.96	-0.27	-3.61	2.91
Baseline	-	-	-	-	-	-
Week 4	-1.42	0.89	66.13	-1.60	-3.17	0.21
Dose by time (Placebo, Baseline)	-	-	-	-	-	-
Dose by time (400mg CBD, Week 4)	-0.27	1.24	66.12	-0.22	-2.59	2.32
Dose by time (800mg CBD, Week 4)	1.07	1.24	65.78	0.86	-1.26	3.46

CI = 95% confidence interval, bootstrapped. SE = Standard Error. Df = Degrees of freedom.

Table S18

Change from baseline to week 4 (end of treatment) for number of drug (cannabis) words recalled, by treatment group.

Contrast	Treatment	Estimate	SE	Df	Lower CI	Upper CI
Week 4 - Baseline	Placebo	-0.71	0.44	65.76	-1.59	0.10
Week 4 - Baseline	400mg CBD	-0.84	0.43	65.73	-1.68	0.05
Week 4 - Baseline	800mg CBD	-0.17	0.44	65.04	-1.06	0.69

CI = 95% confidence interval, bootstrapped. SE = Standard Error. Df = Degrees of freedom.

Figure S7
Group means of number of words recalled on the drug fluency task by treatment group, at baseline and week 4. Error bars represent bootstrapped 95% confidence intervals.

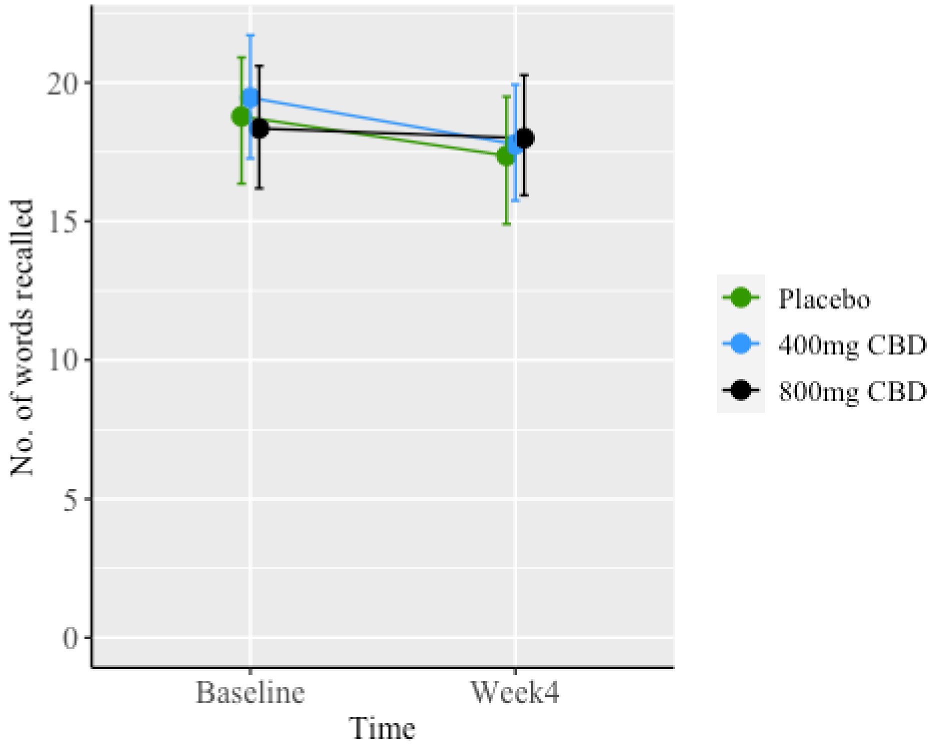

Exploratory Bayes Factors

Table S19
Omnibus Bayes Factors for dose by time interaction in all models with null findings at 95% confidence interval level

Outcome	Bayes Factor
Prose Recall Delayed	0.057
Prose Recall Immediate	0.121
SSRT	0.041
TMT Part A	0.057
TMT Part B – Part A	0.153
Digit Span	0.048
Letter fluency	0.052
Category fluency	0.073
Drug fluency	0.076

Bayes Factors between 0.10-0.05 indicate strong evidence for the null hypothesis (1). Bayes Factors between 0.33–0.10 indicate substantial evidence for the null hypothesis.

1. Jarosz AF, Wiley J. What are the odds? A practical guide to computing and reporting Bayes factors. J Probl Solving. 2014;7(1):2.
